# Late-stage inhibition of autophagy enhances calreticulin surface exposure

**DOI:** 10.18632/oncotarget.13099

**Published:** 2016-11-04

**Authors:** Dan-Dan Li, Bo Xie, Xiao-Jun Wu, Jing-Jing Li, Ya Ding, Xi-Zhi Wen, Xing Zhang, Shu-Guang Zhu, Wei Liu, Xiao-Shi Zhang, Rui-Qing Peng

**Affiliations:** ^1^ Biotherapy Center, Sun Yat-Sen University Cancer Center, State Key Laboratory of Oncology in South China, Collaborative Innovation Center for Cancer Medicine, Guangzhou 510060, China; ^2^ Department of Pharmacology, Zhongshan School of Medicine, Sun Yat-Sen University, Guangzhou 510080, China; ^3^ Department of Colorectal Surgery, Sun Yat-Sen University Cancer Center, State Key Laboratory of Oncology in South China, Collaborative Innovation Center for Cancer Medicine, Guangzhou 510060, China; ^4^ Department of Hepatic Surgery, Liver Transplant Center, Third Affiliated Hospital of Sun Yat-Sen University, TianHe District, Guangzhou 510630, China; ^5^ Guangdong Provincial Key Laboratory of Liver Disease Research, The Third Affiliated Hospital of Sun Yat-Sen University, Guangzhou 510630, China

**Keywords:** calreticulin, autophagy, endoplasmic reticulum stress, mTOR, chemotherapy induced immunogenic cell death

## Abstract

Calreticulin (CRT) exposure on the cell surface is essential for inducing immunogenic cell death by chemotherapy. Recent studies have shown conflicting effects of chemotherapy-induced autophagy on CRT exposure in cancer cells. Our data revealed that surface-exposed CRT (Ecto-CRT) emission was attenuated by inhibition of autophagy at early stages; however, inhibition of autophagy at late stages resulted in increased Ecto-CRT. Furthermore, neither autophagy activation nor endoplasmic reticulum (ER) stress induction alone was sufficient for CRT surface exposure. Moreover, chemotherapeutic agents that only activated autophagy without inducing ER stress could not increase Ecto-CRT; therefore, combined use of an autophagy activator and ER stress inducer could effectively promote CRT translocation to the plasma membrane. Together, our results highlight the potential of the combined use of ER stress inducers and autophagy late-stage inhibitors to reestablish and strengthen both the CRT exposure and immunogenicity of chemotherapeutic agents induced death cells.

## INTRODUCTION

Some chemotherapeutic agents, such as Cyclophosphamide, Doxorubicin, Epirubicin, Mitoxantrone and Oxaliplatin, can induce immunogenic cell death (ICD) of cancer cells [1]; however, clinically these agents fail to lead to tumor rejection thus unaffecting prognosis in patients with malignant disease. Therefore, current chemotherapeutic agents are ineffective at triggering ICD of cancer cells at the patient level.

ICD is characterized by pre-apoptotic exposure of CRT on the cell surface [2], post-apoptotic release of the chromatin-binding protein high mobility group B1 (HMGB1) protein [3, 4], and secretion of adenosine triphosphate (ATP) [5, 6]. Previous reports suggested that autophagy acts as an ‘enabler’ of ICD by assisting in ATP secretion [7], while other studies demonstrated opposing effects of chemotherapy-induced autophagy on CRT exposure in cancer cells. Autophagy was found to suppress the induction of Ecto-CRT [8, 9]; however, it was shown that autophagy-incompetent tumor cells can escape from chemotherapy-induced immunosurveillance [10]. These conflicting observations illustrate that how autophagy participates and regulates chemotherapy-induced CRT exposure remains unclear.

In this study, we found that ER stress was required for autophagy activation during Oxaliplatin treatment. Ecto-CRT emission was attenuated by inhibition of autophagy at early stages, but was increased by inhibition of autophagy at late stages. These observations establish a new combinatorial strategy to improve CRT exposure.

## RESULTS

### Autophagy is essential for oxaliplatin induced CRT surface exposure

**Figure 1:**
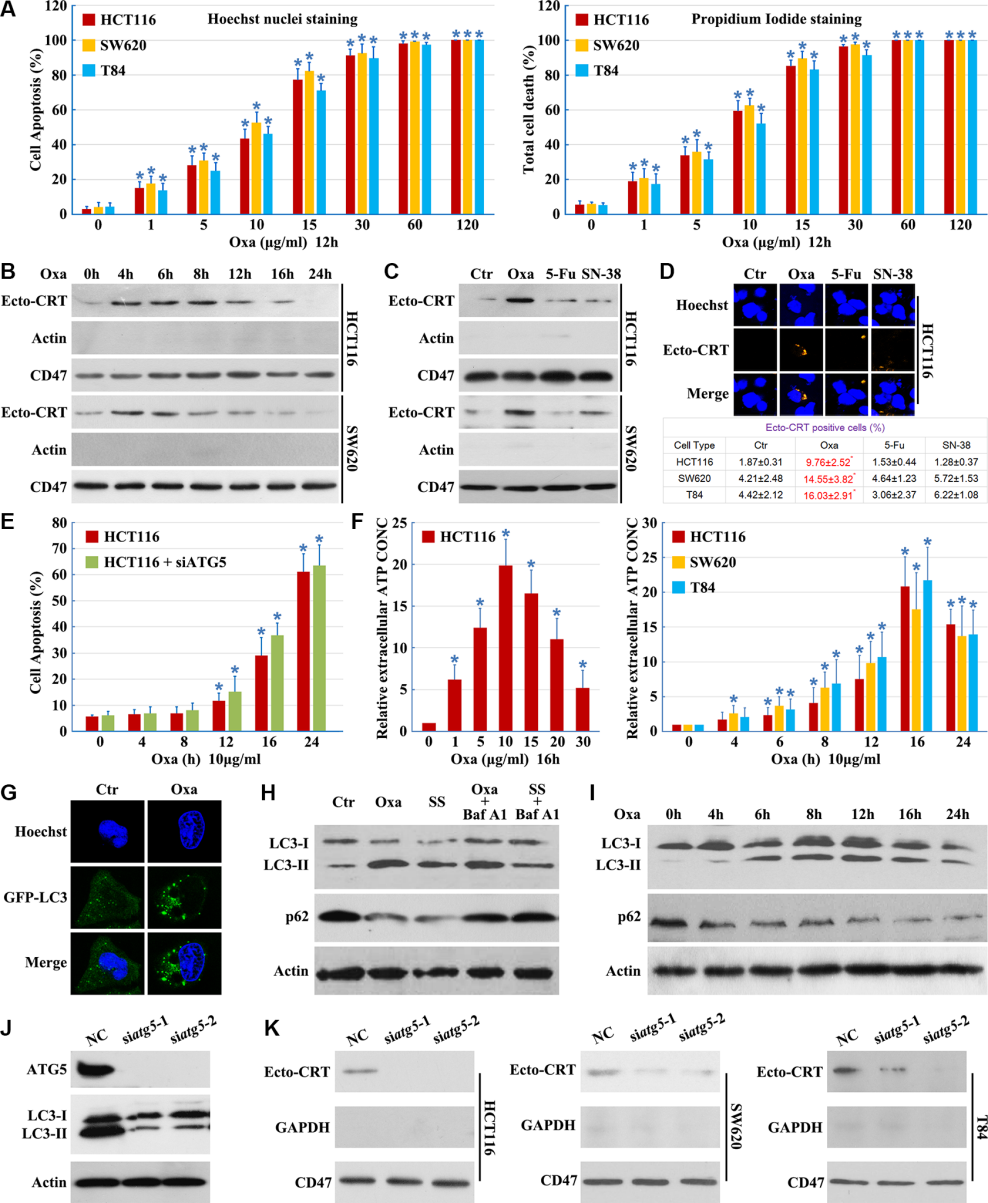
Autophagy is essential for Oxaliplatin induced CRT surface exposure (**A**) Cells were cultured in Oxaliplatin (Oxa) for the indicated concentrations, then apoptosis was determined by the cells had pyknotic nuclei (left) and total cell death was determined by the cells had propidium iodide staining (right); (**B**) Cells were cultured in 10 μg/ml Oxaliplatin (Oxa) for the indicated times, then were followed by purification of the biotinylated plasma membrane proteins and immunoblot detection of Ecto-CRT; (**C**) Cells were treated for 8 h with Oxa 10 μg/ml, 5-Fu 10 μg/ml and SN-38 80 nM. Then were treated as in A; (**D**) Cells were treated as in B, then immunofluorescence detection of Ecto-CRT (upper panel); Ecto-CRT positive cells were quantified (lower panel); (**E**) HCT116 cells were transfected with ATG5 siRNAs for 12 h, then cells were treated with 10 μg/ml Oxa for the indicated times, apoptosis was determined by the cells had pyknotic nuclei; (F left) ATP quantification in HCT116 cell supernatant after a 16 h treatment at indicated concentrations of Oxa; (F right) ATP quantification in cell supernatant after a 10 μg/ml treatment at indicated times of Oxa; (**G**) HCT116 cells were transfected with GFP-LC3 plasmids, then maintained in media with 10 μg/ml Oxa for 12 h. The cells were analyzed by fluorescence microscopy; (**H**) HCT116 cells were treated with 10 μg/ml Oxa for 12 h, serum starvation (SS) for 12 h, 10 μg/ml Oxa for 6 h plus 50 nM Bafilomycin A1 (Baf A1) 6 h or SS for 6 h plus 50 nM Baf A1 6 h, then cells were subjected to immunoblot detection; (**I**) HCT116 cells were treated with 10 μg/ml Oxa for indicated times, then cells were subjected to immunoblot detection; (**J**–**K**) Cells were transfected with ATG5 siRNAs for 12 h, then maintained in media with 10 μg/ml Oxa for 12 h (J) or 8 h (K), then cells were subjected to immunoblot detection (J) or Ecto-CRT detection (K). Results are representative of three independent experiments. The values represent the mean ± S.E. of at least three independent experiments. * denotes *p* < 0.05.

Treatment of a series of colon cancer cell lines with Oxaliplatin induces apoptosis (Figure 1A) and stimulates pre-apoptotic CRT exposure [11]. We measured exposed CRT on the surface of human colon cancer cells after stimulation with Oxaliplatin (Figure 1B–1D). CRT surface exposure preceded Oxaliplatin-induced cell apoptosis (Figure 1E); however, 5-Fluorouracil (5-Fu) and SN-38 (the active metabolite of irinotecan, an analog of Camptothecin [12]) failed to induce pre-apoptotic CRT exposure (Figures 1C and 1D). Additionally, Oxaliplatin treatment induced the release of more ATP from the cells (Figure 1F).

Furthermore, we compared the autophagic activity of control cells and Oxaliplatin treated cells using a GFP-LC3 light microscopy assay [13]. We observed an accumulation of punctate GFP-LC3 staining following Oxaliplatin treatment, suggesting the induction of autophagy (Figure 1G). Treatment with Oxaliplatin or serum starvation resulted in an increase of LC3-II and a decrease of p62, with ablation of autophagy by Bafilomycin A1 (Figures 1H and 1I). These results illustrate that Oxaliplatin induces a complete autophagic response.

To determine whether autophagy is involved in CRT plasma membrane translocation, cells were transfected with ATG5 siRNAs. Levels of ATG5 were effectively reduced by ATG5 siRNAs and Oxaliplatin-induced autophagy was blocked (Figure 1J). Knockdown of ATG5 did not enhance Oxaliplatin-induced cell apoptosis (Figure 1E), demonstrating the efficacy and specificity of the siRNAs. We observed a significant decrease in Ecto-CRT emission upon ATG5 knockdown (Figure 1K). These results indicate that autophagy is essential for Oxaliplatin-induced CRT surface exposure.

### Beclin 1 is required but not sufficient for CRT surface exposure

**Figure 2:**
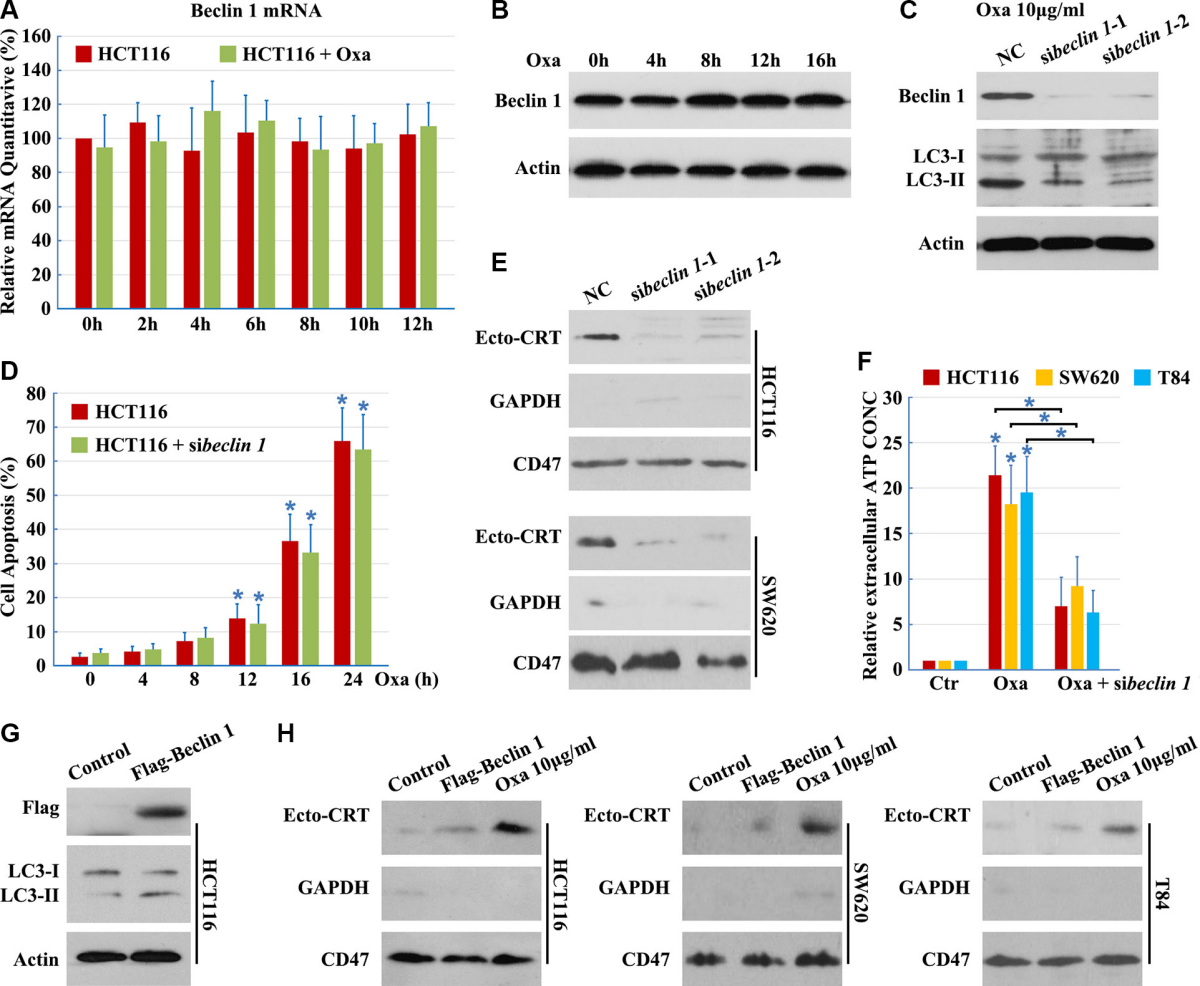
Beclin 1 is required but not sufficient for CRT surface exposure (**A**–**B**) HCT116 cells were treated with 10 μg/ml Oxaliplatin (Oxa) for indicated times, total RNA was extracted and analyzed by Q-PCR (A), total protein was extracted and analyzed by Western blotting (B); (**C**) HCT116 cells were transfected with Beclin 1 siRNAs for 12 h, then maintained in media with 10 μg/ml Oxa for 12 h. Then cells were subjected to immunoblot detection; (**D**) HCT116 cells were transfected with Beclin 1 siRNAs for 12 h, then cells were treated with 10 μg/ml Oxa for the indicated times. Apoptosis was determined by the cells had pyknotic nuclei; (**E**) Cells were treated as in C for 8 h. Then cells were subjected to Ecto-CRT detection; (**F**) ATP quantification in cell supernatant after a 16 h treatment with 10 μg/ml Oxa or Oxa plus Beclin 1 siRNA; (**G**–**H**) Cells were transfected with Flag-Beclin 1 expression plasmid for 12 h, then maintained in media with (G) or without (H) 10 μg/ml Oxa for 8 h. The cells were subjected to immunoblot detection (G) or Ecto-CRT detection (H). Results are representative of three independent experiments. The values represent the mean ± S.E. of at least three independent experiments.

Beclin 1 complexes with class III PI3K and is required for autophagy under conditions of nutrient starvation [14]. Thus, we examined the role of Beclin 1 in Oxaliplatin-induced autophagic cell death. However, Oxaliplatin did not elicit an increase in Beclin 1 expression (Figure 2A and 2B).

To determine whether Beclin 1 is involved in autophagy, we designed siRNAs to reduce human Beclin 1. Beclin 1 mRNA was effectively targeted by introduction of siRNAs, reducing Beclin 1 protein levels and blocking Oxaliplatin-induced autophagy (Figure 2C), while not enhancing Oxaliplatin-induced cell apoptosis (Figure 2D), demonstrating the efficacy and specificity of the Beclin 1 siRNAs. In addition, there was a significant decrease in Ecto-CRT emission and ATP secretion after Beclin 1 knockdown (Figure 2E and 2F), indicating that Beclin 1 is required for Oxaliplatin-induced CRT surface exposure.

Furthermore, to determine whether Beclin 1 was sufficient to promote Ecto-CRT emission, wild-type Beclin 1 was transfected into cells. Overexpression of wild-type Beclin 1 increased the levels of LC3-II (Figure 2G), suggesting that Beclin 1 promotes cellular autophagic flux. However, wild-type Beclin 1 overexpression did not increase the induction of Ecto-CRT (Figure 2H). Thus, Beclin 1 is necessary, but not sufficient for Oxaliplatin-induced CRT surface exposure.

### Oxaliplatin-induced mTOR-dependent autophagy is required for CRT surface exposure

**Figure 3:**
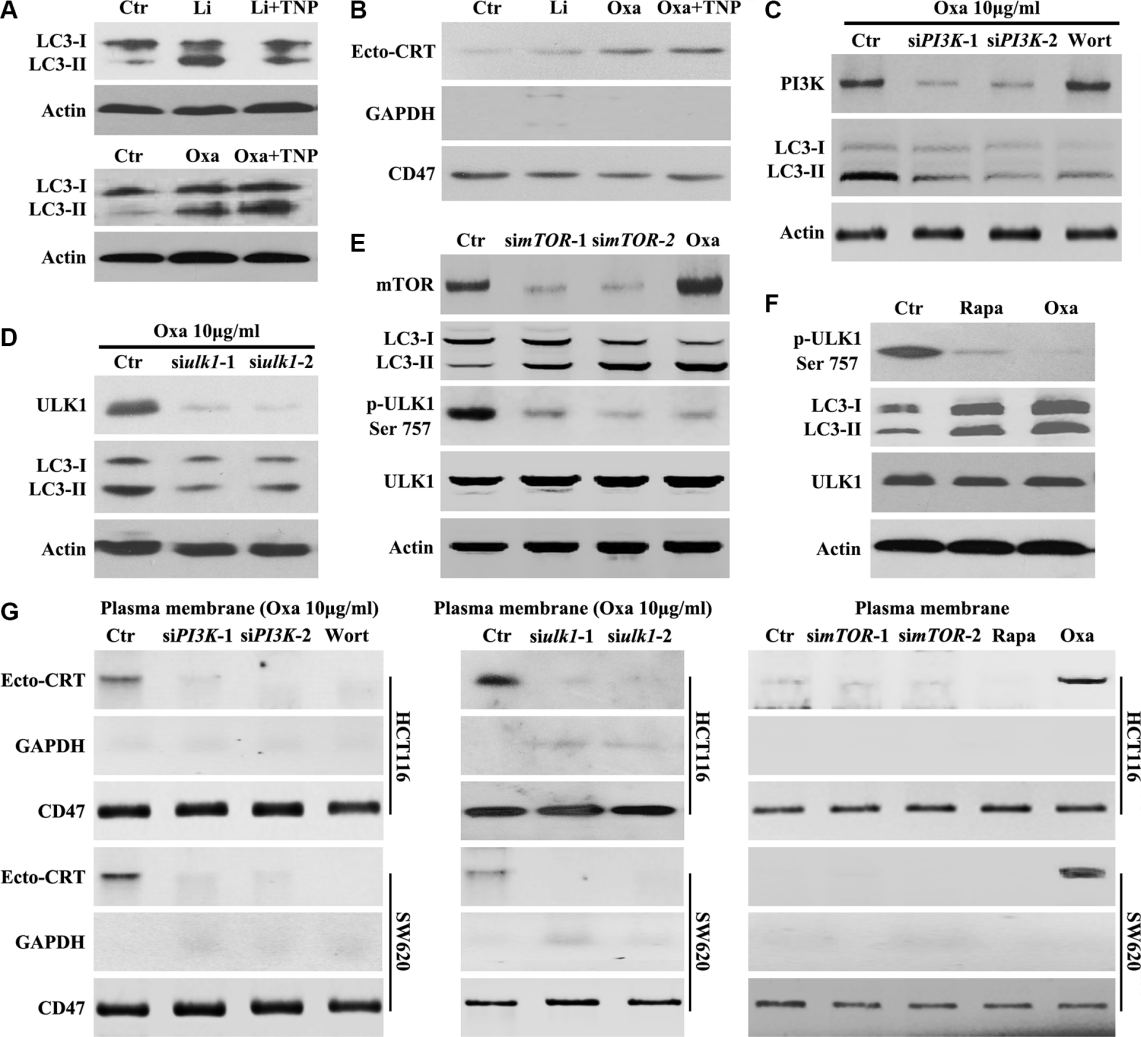
Oxaliplatin induced mTOR-dependent autophagy is required for CRT surface exposure (**A**–**B**) HCT116 Cells were treated for 8 h with Oxaliplatin (Oxa) 10 μg/ml or Lithium (Li) 10 mM and with or without TNP 20 μM, then subjected to immunoblot detection of LC3 in the total cell lysate (A) or Ecto-CRT detection (B); (**C**) HCT116 Cells were treated with 10 μg/ml Oxa, Oxa plus PI3K siRNA or Oxa plus 50 nM Wortmannin (Wort) for 8 h, then subjected to immunoblot detection; (**D**) HCT116 Cells were treated with 10 μg/ml Oxa or Oxa plus PI3K siRNA for 8 h, then subjected to immunoblot detection; (**E**) HCT116 Cells were treated with mTOR siRNA for 24 h or 10 μg/ml Oxa for 8 h, then subjected to immunoblot detection; (**F**) HCT116 cells were treated with 10 μg/ml Oxa or Rapamycin (Rapa) 100 nM for 8 h, then cells were subjected to immunoblot detection; (**G** left) Cells were transfected with or without PI3K siRNAs for 12 h, then maintained in media with 10 μg/ml Oxa or 50 nMWort for 8 h, then subjected to Ecto-CRT detection; (G middle) Cells were transfected with or without ULK1 siRNAs for 12 h, then maintained in media with 10 μg/ml Oxa for 8 h, then subjected to Ecto-CRT detection; (G right) Cells were transfected with or without ULK1 siRNAs for 24 h or treated with 10 μg/ml Oxa or 100 nM Rapa for 8 h, then subjected to Ecto-CRT detection. Results are representative of three independent experiments.

Several signaling pathways are known to regulate autophagy [15–17], either through mTOR dependent or independent mechanisms. Activation of autophagy through a mTOR-dependent pathway requires the activation of ULK1 [17], while levels of Inositol-1,4, 5-Triphosphate (IP3), activated by lithium [18], trigger autophagy through a mTOR-independent mechanism. To determine whether Oxaliplatin-induced autophagy is mTOR-independent, TNP [N2-(m-trifluorobenzyl),N6-(p-nitrobenzyl) purine, a membrane-permeable inhibitor of IP3-3K [19] was employed. We confirmed that TNP counteracted lithium, but not Oxaliplatin-induced increase of LC3-II (Figure 3A). Lithium could not promote CRT translocation and TNP had no effect on Oxaliplatin-induced CRT translocation (Figure 3B). Conversely, cells treated with PI3K siRNAs, Wortmannin (PI3K inhibitor), or ULK1 siRNAs were able to inhibit Oxaliplatin-induced autophagy (Figure 3C and 3D). Furthermore, ULK1 Ser757 phosphorylation, an indicator of high mTOR activity [20], was inhibited in cells treated with mTOR siRNAs, Rapamycin (mTOR inhibitor) or Oxaliplatin (Figures 3E and 3F), confirming that Oxaliplatin-induced autophagy is mediated by the mTOR/ULK1 pathway (Figure 3D).

Further investigation demonstrated that Oxaliplatin was unable to induce CRT surface exposure in cells treated with PI3K siRNAs, Wortmannin, or ULK1 siRNAs (Figure 3G). Additionally, mTOR siRNAs or Rapamycin could not promote CRT translocation (Figure 3G). Taken together, these results suggest that autophagy and CRT surface exposure observed upon Oxaliplatin treatment is mediated through the PI3K/mTOR-dependent pathway.

### Oxaliplatin promotes beclin 1 phosphorylation by enhancing the association with ULK1

**Figure 4:**
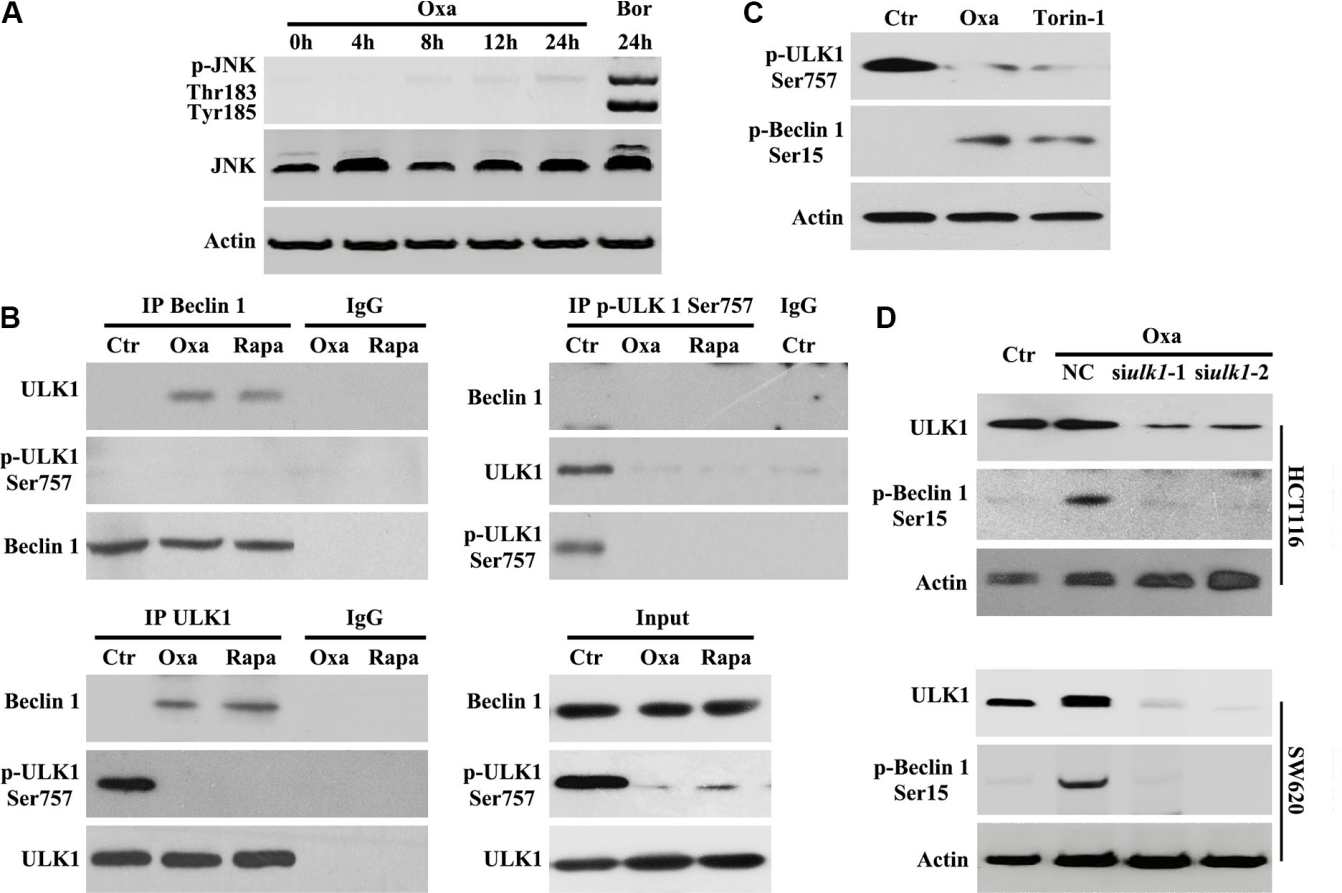
Oxaliplatin promotes Beclin 1 phosphorylation by enhancing the association with ULK1 (**A**) HCT116 cells were treated with 10 μg/ml Oxaliplatin (Oxa) for indicated times or 50 nM Bortezomib (Bor) for 24 h, then subjected to immunoblot detection; (**B**) HCT116 cells were treated with 10 μg/ml Oxaliplatin (Oxa) or 100 nM Rapamycin (Rapa) for 8 h were lysed and subjected to immunoprecipitation analysis using an indicated antibody, then immunoprecipitates were subjected to immunoblot detection; (**C**) HCT116 cells were treated with 1 μg/ml Oxa or 250 nM Torin-1 for 8 h were lysed and subjected to immunoblot detection; (**D**) Cells were transfected with ULK 1 siRNAs for 12 h and maintained in media with 1 μg/ml Oxa for 8 h, then were followed by immunoblot detection. Results are representative of three independent experiments.

Beclin 1 is an integrator of autophagic signaling [21]. Activated Caspase 8 was reported to cleave Beclin 1 and suppresse Beclin 1 associated autophagy and enhanced apoptosis [22]. In our study, a time-course analysis of Beclin 1 using an antibody anti-1-300AA (from Santa Cruz Bio., Cat No. sc-11427) of Beclin 1 did not show any truncated isoforms of Beclin 1 (Figure 2B). Oxaliplatin and Bortezomib have been shown to activate the JNK-Bcl-xL-Bax pathway leading to the dissociation of Bcl-xL from Beclin-1, subsequently activating autophagy [22]. Consistent with previous reports [22], we found that Oxaliplatin alone was unable to activate JNK (Figure 4A). Therefore, Beclin 1 activity is not modulated by Caspase-8 or JNK pathway during Oxaliplatin treatment.

ULK1 directly phosphorylates Beclin 1 and activates hVPS34 in response to mTOR inhibition [23]. Next, we determined whether Beclin 1 activation was mediated by ULK1 during Oxaliplatin-induced autophagy. We performed immunoprecipitation assays to determine the interaction between Beclin 1 and ULK1. In HCT116 cells treated with Oxaliplatin or Rapamycin, we observed Beclin 1 coimmunoprecipitated with unphosphorylated Ser757 ULK1 (Figure 4B). Importantly, phosphorylated Ser757 ULK1 could not immunoprecipitate Beclin 1 following Oxaliplatin or Rapamycin treatment (Figure 4B), suggesting that Oxaliplatin treatment may promote Beclin1/ULK1 interaction necessary for phosphorylation.

To confirm the role of mTOR in Beclin 1 activation, western blot analysis of Beclin 1 phosphorylation was performed following Oxaliplatin or torin-1 (mTOR inhibitor [25]) treatment and showed that endogenous Beclin 1 was phosphorylated at Ser15 (Ser14 in mouse Beclin 1 [24]) (Figure 4C). To determine if ULK1 was required for Beclin 1 phosphorylation, HCT116 cells were treated with ULK1 siRNA and Oxaliplatin. Inhibition of ULK1 resulted in a clear downregulation of Beclin 1 Ser15 phosphorylation during Oxaliplatin treatment (Figure 4D), implicating ULK1 in Beclin 1 phosphorylation.

### Inhibition of autophagy at different stages affects CRT exposure

The process of autophagy is divided into five steps: initiation, elongation, maturation, fusion and degradation, which are controlled by a set of autophagy-related genes (ATGs) [13, 26, 27]. While the function of autophagy in CRT translocation is controversial, we speculate that different stages of autophagy may play different roles in the translocation of CRT. Attenuation of autophagy at early stages, through siRNA silencing of ULK1 and Beclin 1 or ATG5 expression, resulted in significantly decreased levels of Ecto-CRT (Figures 1J, 2E and 3G).

**Figure 5:**
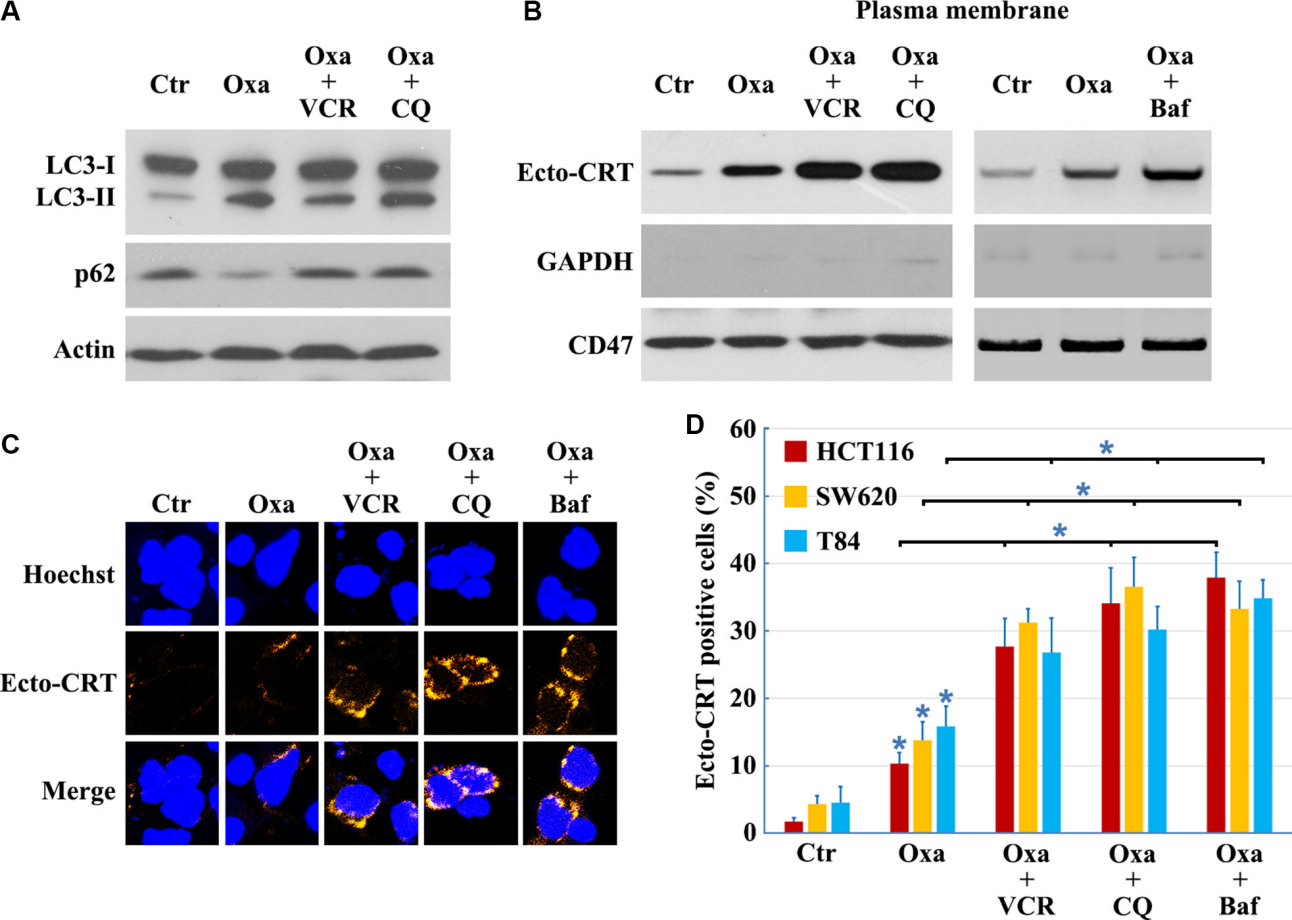
Inhibition of autophagy at early stage decreases, but at late stage increases CRT exposure Cells treated with 10 μg/ml Oxaliplatin (Oxa) 8 h, 10 μg/ml Oxa 2 h plus 50 nM VCR 6 h, 10 μg/ml Oxa 2 h plus 50 μM CQ 6 h or 10 μg/ml Oxa 2 h plus 50 nM Bafilomycin A1 (Baf) 6 h, then were lysed and subjected to immunoblot detection (**A**), or subjected to Ecto-CRT detection (**B**), or send to immunofluorescence detection of Ecto-CRT (**C**), and Ecto-CRT positive cells were quantified (**D**). Results are representative of three independent experiments. The values represent the mean ± S.E. of at least three independent experiments. * denotes *p* < 0.05.

Compared to early stages of autophagy, relatively little is known about the later stages of autophagy; however, recent work had demonstrated that suppression of autophagy at late stages could efficiently augment anticancer therapy-induced cytotoxicity [28]. We used vincristine (VCR, inhibits autophagy at a late stage by blocking fusion of autophagosomes with lysosomes [29]), CQ (induces lysosomal disruption to inhibit autophagy at a relatively late stage [29]) and Bafilomycin A1 (prevents maturation of autophagic vacuoles by inhibiting fusion between autophagosomes and lysosomes) to study the influence of late stage inhibition of autophagy on CRT translocation. VCR, CQ and Bafilomycin A1 prevented the Oxaliplatin-induced reduction in p62 protein levels (Figures 1G and 5A), consistent with an inhibition of autophagy. In addition, LC3B-II protein levels were not attenuated in cells treated with Oxaliplatin following addition of VCR, CQ or Bafilomycin A1 (Figures 1G and 5A), confirming that VCR, CQ and Bafilomycin A1 inhibit autophagy at a late stage. Moreover, inhibition of autophagy at late stages had a significant effect on Ecto-CRT exposure, compared to cells treated by Oxaliplatin only (Figure 5B–5D). Together, our findings suggest that CRT exposure is attenuated by inhibition of autophagy at early stages, but augmented by inhibition of autophagy at late stages.

### Combined use of an ER stress inducer and an autophagy late stage inhibitor restores the ICD inducer activity of 5-Fu and SN-38

**Figure 6:**
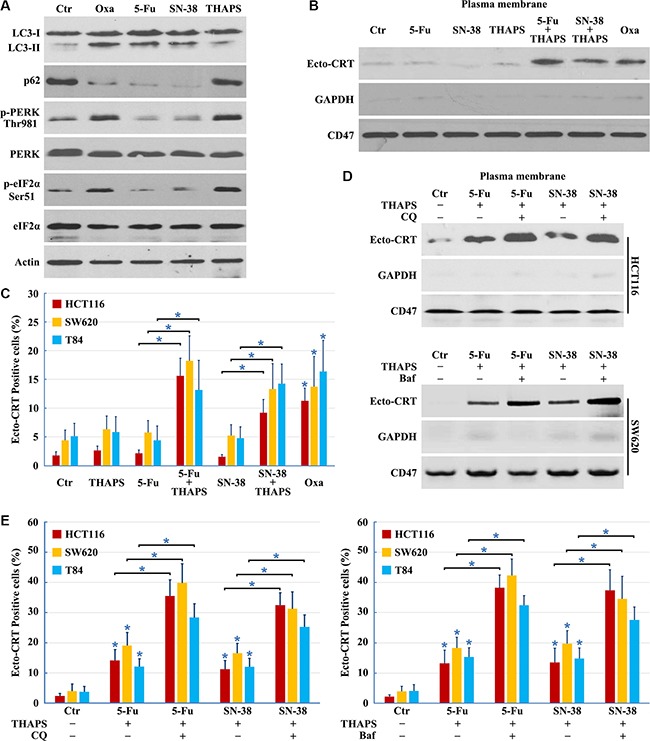
Combined use of ER stress inducer and autophagy late stage inhibitor to restore the ICD inducer activity of 5-Fu and SN-38 (**A**) HCT116 cells treated with 10 μg/ml Oxaliplatin (Oxa), 10 μg/ml 5-Fu, 80 nM SN-38 or 500 nM THAPS for 8 h were lysed and subjected to immunoblot detection; (**B**–**C**) Cells were treated with 10 μg/ml Oxa, 10 μg/ml 5-Fu, 80 nM SN-38, 500 nM THAPS, 10 μg/ml 5-Fu plus 500 nM THAPS or 80 nM SN-38 plus 500 nM THAPS for 8 h followed by Ecto-CRT detection (B) or send to immunofluorescence detection and quantification of Ecto-CRT (C); (**D**–**E**) Cells were treated with 10 μg/ml 5-Fu plus 500 nM THAPS, 80 nM SN-38 plus 500 nM THAPS, 10 μg/ml 5-Fu plus 500 nM THAPS plus 50 μM CQ/ 50 nM Bafilomycin A1 (Baf) or 80 nM SN-38 plus 500 nM THAPS plus 50 μM CQ/ 50 nM Baf for 8 h followed by Ecto-CRT detection (D) or send to immunofluorescence detection of Ecto-CRT (E). Results are representative of three independent experiments. The values represent the mean ± S.E. of at least three independent experiments. * denotes *p* < 0.05.

Several anticancer drugs, including 5-Fu, camptothecin, cisplatin, docetaxel, etoposide, mitoxantrone, doxorubicin, and Oxaliplatin among others, can induce autophagy; however, only a fraction of drugs, such as mitoxantrone, doxorubicin and Oxaliplatin, can promote CRT translocation from the ER lumen to the surface of stressed and dying cancer cells, inducing *bona fide* ICD [1]. Previous studies indicated that the incapacity of a drug to elicit translocation of CRT from the lumen of the ER [30] is attributed to the failure to induce ICD. While Oxaliplatin, 5-Fu, and SN-38 increased LC3-II and decreased p62 levels indicating successful autophagy induction (Figure 6A), neither 5-Fu nor SN-38 was able to induce ER stress (Figure 6A). Both 5-Fu and SN-38 also failed to trigger PERK-dependent phosphorylation of eIF2α (Figure 6A). Thapsigargin (THAPS), an inhibitor of the Sarco/ER Ca^2+^-ATPase that does not stimulate autophagy [30, 31], was used to induce ER stress in 5-Fu or SN-38 treated colon cancer cells (Figure 6A). Although THAPS, 5-Fu or SN-38 individually was insufficient to induce CRT exposure, the combination of THAPS and 5-Fu or SN-38 effectively prompted the translocation of CRT to the plasma membrane (Figure 6B and 6C). Our results illustrate that ER stress and autophagy are required for CRT exposure, and the combined use of ER stress inducers can establish the immunogenicity of 5-Fu or SN-38-induced death cancer cells.

Although ER stress induction by THAPS could stimulate CRT exposure when combined with 5-Fu or SN-38 treatment, it failed to induce the massive CRT exposure necessary for immunogenic cell death; therefore, we determined whether addition of the autophagy late stage inhibitor could improve CRT exposure in 5-Fu or SN-38 treated cells. We found that inhibition of autophagy at late stages with CQ or Bafilomycin A1 significantly improved CRT surface exposure, compared to the cells treated by THAPS/5-Fu or THAPS/5SN-38 (Figure 6D and 6E). In conclusion, the combined use of an ER stress inducer and autophagy late stage inhibitor can enhance the expression of Ecto-CRT in 5-Fu or SN-38 treated cells.

## DISCUSSION

Cellular pre-mortem autophagy is important for the release of danger-associated molecular patterns and antigens. Hence, stimulation of autophagy is an important prerequisite for ICD in cancer cells [5]. Consequently, autophagy-deficient tumor cells treated with ICD inducers *in vitro* fail to induce a tumor-specific immune response *in vivo* because of the inability to release ATP [10], thus enhanced autophagy correlates with activation of anti-tumor immunity and its downregulation may contribute to immune system avoidance in malignant growths. Surprisingly, it was reported that autophagy suppresses the induction of Ecto-CRT [8, 9], appearing to act as an ICD inhibitor. In this study, we found that early stage inhibition of autophagy attenuated CRT exposure. The use of Oxaliplatin at differing concentrations may contribute to these disparate findings. Data from clinical trials showed that the maximal blood Oxaliplatin concentration was 1.44 ± 0.20 (SD) μg/mL (3.6 ± 0.50 μM) when patients were given 85 mg/m^2^ of Oxaliplatin using an infusion time of 2 h [32]. The Oxaliplatin concentrations used by Michaud et al. were 75, 150 and 300 μM; whereas our group more closely recapitulated biological levels using only 10 μg/ml (25 μM). We found the IC50 of Oxaliplatin for HCT116 cells was 25 μM. Higher concentrations of Oxaliplatin (≥ 30 μg/ml or ≥ 75 μM) induced quick apoptosis (< 12 h) and resulted in a higher apoptotic rate (> 96%) of colon cancer cells. Importantly, this level of apoptosis activated caspases or calpains, which digest several essential autophagy proteins (such as Atg3, Beclin 1 and AMBRA1), thereby disrupting the normal autophagic program [33–35]. We hypothesized that early stage autophagy activated by low Oxaliplatin dosage may promote CRT exposure; however, activation from high Oxaliplatin dosage may inhibit CRT exposure. The exact mechanisms need to be further explored.

We demonstrate here that mTOR-dependent autophagy is required for Oxaliplatin-induced CRT surface exposure. Interestingly, we observed that autophagy alone was not sufficient to promote CRT translocation. As the ER is the central intracellular organelle in the secretory pathway and the ER stress response elicited by chemotherapeutic agents participates in the CRT exposure pathway [2, 36–38], we further demonstrated that Oxaliplatin-induced autophagy was dependent on ER stress. Moreover, THAPS, an ER stress inducer, can restore CRT exposure and 5-Fu or SN-38-induced cancer cell death (5-Fu or SN-38 induce autophagy but not ER stress). Together, these findings suggest that both ER stress and autophagy are required for immunogenicity and drug-triggered ER stress can restore the immunogenicity of anticancer drugs that induce autophagy without induction of ER stress.

We found that Oxaliplatin treatment, which effectively induces ER stress, combined treatment with an ER stress inducer and 5-Fu or SN-38 failed to induce massive CRT exposure, with only 10–15% of treated cells exposing CRT. To date, there are no strategies proven to boost Ecto-CRT. As we found autophagy was required for chemotherapy-induced CRT exposure, we hypothesized that manipulation of the autophagic response could efficiently augment efficacy of CRT exposure on those treated cells, and thus we examined the effect of differential inhibition of autophagy on Oxaliplatin-induced CRT exposure. Our western blot results are consistent with the model that siRNA silencing of ULK1, Beclin 1 or ATG5 inhibits cells from CRT exposure induced by chemotherapeutic agents. Using VCR, CQ and Bafilomycin A1 to inhibit autophagy at late stages significantly improved CRT surface exposure, compared to the cells treated with Oxaliplatin alone. Likewise, combined use of an autophagy late stage inhibitor also augmented the ability of THAPS to restore 5-Fu or SN-38-induced CRT exposure. Our study suggests that CRT exposure is attenuated by inhibition of autophagy at early stages, but augmented by inhibition of autophagy at late stages, and these novel findings are consistent with previous reports that inhibition of autophagy at late stages can effectively augment cytotoxicity of anticancer therapy in human malignant tumor cells [28, 29]. Taken together, CRT exposure is dependent on the type of autophagic response elicited by the type of autophagy inhibitor. Thus, optimized modulation of autophagy is essential for enhancing the efficacy of chemotherapy induced ICD.

Although further studies are essential to elucidate the molecular mechanisms of autophagy that guide CRT translocate from ER to the plasma membrane, our results highlight the potential for the use of combined ER stress inducers and autophagy late stage inhibitors to reestablish or strengthen the immunogenicity of chemotherapeutic agents induced death cancer cells.

## MATERIALS AND METHODS

### Cell culture

HCT116, SW620 and T84 human colon cancer cells were obtained from the American Type Culture Collection (ATCC) (Manassas, VA, USA) and were cultured at 37°C in 5% CO_2_ in Dulbecco's modified Eagle's medium (DMEM) (Invitrogen) supplemented with 1% penicillin (100 U/ml, Invitrogen), 1% streptomycin (100 μg/ml, Invitrogen), L-glutamine (292 μg/ml, Invitrogen) and 10% fetal bovine serum (FBS; hyClone Laboratories).

### Reagents

Oxaliplatin (Sigma), Lithium chloride (Sigma), 5-Fluorouracil (Sigma-Aldrich), Hoechst 33258 (Sigma-Aldrich), SN-38 (Santa Cruz Biotechnology), N2-(m-trifluorobenzyl),N6-(p-nitrobenzyl)purine (Sigma-Aldrich), Wortmannin (Selleck Chemicals), Rapamycine (Selleck Chemicals), Torin-1 (Selleck Chemicals), Bafilomycin A1 (Selleck Chemicals), GSK2656157 (Selleck Chemicals), Vincristine (Selleck Chemicals), Chloroquine (Selleck Chemicals), Thapsigargin (Sigma) were added to the media at the indicated concentrations and time points. Lipofectamine 2000 (Invitrogen) was used for transient gene or siRNA transfection of cells.

The following primary antibodies were used: Calreticulin (#ab2907 and #ab22683) and CD47 (#ab108415) were from Abcam plc.; ULK1 (#8054), phospho-ULK1 (Ser757) (#6888), p62 (#8025), Beclin 1 (#3738), phosphor-Beclin 1 (Ser15) (#13825), ATG5 (#2630), eIF2α (#2103) and phospho-eIF2α (Ser51) (#3597) were from Cell Signaling Technology; Beclin 1(#sc-11427), β-Actin (#sc-130656), GAPDH (#sc-47724), PERK (#sc-13073) and phospho-PERK (Thr981) (#sc-32577) were from Santa Cruz Biotechnology; Flag (#F1804) was from Sigma; LC3 (#NB100-2220) was from Novus Biologicals.

### Plasmids

The overexpression plasmids Flag-Beclin 1 and GFP-LC3 were kindly provided by Beth Levine [13].

### Autophagy assays

Autophagy was measured by light microscopic quantitation of cells transfected with GFP-LC3 as described or by Western blotting analysis of the levels of LC3 and p62 [13].

### Immunoblot analysis

Equal amounts of protein (40–50 mg) were size-fractionated using 6–15% SDS-PAGE gradient gels. The resolved proteins were electrophoretically transferred onto polyvinylidene difluoride membranes and analyzed by immunoblotting using an ECL chemiluminescence reagent and XAR film (Kodak, XBT-1) according to the manufacturer's protocol. Primary antibodies were used at optimized dilutions along with the appropriate HRP-conjugated secondary antibodies. The data were collected from at least three independent experiments.

### Immunoprecipitation and immunoblot analysis

cells were grown on 10 cm plates, treated as indicated, washed two times with PBS, and harvested with 1 ml of the following lysis buffer: 20 mM Tris HCl (pH 7.5), 150 mM NaCl, 10% glycerol, 1% Triton X-100, 2 mM EDTA, with complete, EDTA-free protease inhibitor (Roche) and phosphatase inhibitors (Sigma). Cells were left on ice for 20 min and centrifuged at 12,000 rpm for 15 min. Three milligrams of protein lysate were used for immunoprecipitation using 2 mg of the indicated antibodies overnight at 4°C. The following day, 25 μl of protein A/G ultralink resin (Thermo Scientific) was added for 2 h at 4°C. The IPs were washed three times with lysis buffer, then sample buffer was added and the beads were boiled for 5 min at 95°C. The samples were then analyzed by SDS-PAGE followed by immunoblotting with the indicated antibodies.

### Cell surface immunofluorescence

Cells were grown on 13 mm round glass coverslips. After treatment as described, cells were placed on ice, washed twice with PBS and fixed with 0.37% PFA in phosphate-buffered saline (PBS) for 10min. Cells were then washed twice in PBS and treated by cold blocking buffer for 1 h. After sequential treatment with NH_4_Cl (50 mM in 20 mM glycine) for 10 min, the indicated antibody (1:200 in bovine serum albumin) was added and incubated overnight at 4°C. After an additional incubation for 1 h at room temperature with Hoechst 33258 and fluorescein isothiocyanate-conjugated secondary antibody (Invitrogen) (1:400 in bovine serum albumin), the slides were mounted in anti-fading solution (Permafluor, Beckman Coulter, Krefeld, Germany) and stored at 4°C, followed by confocal laser-scanning microscopy.

### Biotinylation of cell surface proteins

Biotinylation and recovery of cell surface proteins were performed with a reported method [2]. Briefly, 20 × 10^6^ HCT116 cells grown on 75 cm^2^ flask were placed on ice and washed three times with ice-cold PBS-Ca^2+^-Mg^2+^ (PBS with 0.1 mM CaCl_2_ and 1 mM MgCl_2_). Membrane proteins were then biotinylated by a 30 min incubation at 4°C with NHS-SS-biotin 1.25 mg/ml (Pierce) freshly diluted into biotinylation buffer (10 mM triethanolamine, 2 mM CaCl_2_, 150 mM NaCl, pH 7.5) with gentle agitation. Cells were rinsed with PBS-Ca^2+^-Mg^2+^ plus glycine (100 mM) and washed in this buffer for 20 min at 4°C to quench unreacted biotin. The cells were then rinsed twice with PBS-Ca^2+^-Mg^2+^, scraped in cold PBS, and pelleted at 2,000 rpm at 4°C. The pellets were solubilized for 45 min in 500 μl of lysis buffer (1% Triton X-100, 150 mM NaCl, 5 mM EDTA, 50 mM Tris, pH 7.5) containing protease inhibitors. The lysates were clarified by centrifugation at 14,000 × g for 10 min at 4°C, and the supernatants were incubated overnight with packed streptavidin-agarose beads to recover biotinylated proteins. The beads were then pelleted by centrifugation, and aliquots of supernatants were taken to represent the unbound, intracellular pool of proteins. Biotinylated proteins were eluted from the beads by heating to 100°C for 5 min in SDS-PAGE sample buffer before loading onto a 10% SDS-PAGE gel. To ensure the absence of leakage of biotin into the cells, we systematically verified the absence of the intracellular protein Actin and GAPDH in biotinylated extracts.

### ATP release assays *in vitro*

Extracellular and intracellular ATP levels were measured by the luciferin-based ENLITEN^®^ ATP Assay (Promega) kits, respectively, in excess of luciferin and luciferase, as indicated by the manufacturer. ATP-driven chemoluminescence was recorded on Monolight 3010 Luminometer (Pharmingen).

### siRNA interference

The target sequence for human ATG5-specific siRNAs were 5′-GCAACUCUGGAUGGGAUUG-3′ and 5′-GCAACTCTGGATGGGATTG-3′, for human Beclin 1-specific siRNAs were 5′UGGAAUGGAAUGA GAUUAATT-3′ and 5′-AAGAUUGAAGACACAGGA GGC-3GACACAGGAGGCAUUAATT-3; for human ULK1-specific siRNAs were 5′-GUGGCCCUGUACGAC UUCCAGGAAA-3′ and 5′- GGAGAAAACUUGTAG GUGU-3′, for human mTOR-specific siRNAs were 5′-AAGAAUCAAAGAGCAGAGUGC-3′ and 5′-CAGG CCTATGGTCGAGA TTTA-3′; human PI3K-specific siRNAs were from Dharmacon (ONTARGETplus SMARTpool) and Santacruz, all of which and the negative control siRNA (no silencing small RNA fragment) were synthesized by GenChem Co. (Shanghai, China).

### Quantitative polymerase chain reaction (Q-PCR)

Total RNA was extracted and isolated from cells using TRIzol reagent (Invitrogen) as described previously. First strand cDNA was synthesized from 1 μg of mRNA using Superscript III reverse transcriptase (Invitrogen) and oligo (dT) as primers. Q-PCR was performed in triplicate on an ABI Prism 7000 sequence detection system using an ABI SYBR Green PCR mixture as described by the manufacturer. PCR cycling conditions were as follows: initial denaturation at 95°C for 5–10 min followed by 40 cycles of 95°C for 30 sec, 1 min of annealing, and 1 min of extension at 72°C. The annealing temperature was adapted for the specific primer set used. Fluorescence data were collected during the annealing stage of amplification and specificity of the amplification was verified by melting curve analysis. Cycle threshold (Ct) values were calculated using identical threshold values for all experiments. *gapdh* was used as a control and for normalization. Relative RNA expression was calculated using the formula ratio = 2 (Ctref-Cttarget). Data shown represent the mean and S.E. of three separate experiments. The following primer pairs for *beclin 1* were used: forward (5′-CCAGGCGAAACCAGGAGAGG CTCAGGAGGAAGAGACTAAC-3′) and reverse (5′-GTT AGTCTCTTCCTCCTGAGCCTCTCCTGGTTTCGCCT GG-3′); primer pairs for *gapdh* were used: forward (5′-AAGCCTGCCGGTGACTAAC-3′) and reverse (5′-GTTAAAAGCAGCCCTGGTGAC-3′).

### Immunoprecipitation and kinase assays

Immunoprecipitation experiments and kinase assays were performed as described previously. HEK293T cell extracts were harvested from a 10 cm plate and used for each immunoprecipitation condition. The cells were lysed on ice for 20 min in lysis buffer (40 mM HEPES, pH 7.5, 120 mM NaCl, 0.3% CHAPS, 1 mM EDTA, 10 mM pyrophosphate, 10 mM glycerophosphate, 50 mM NaF, and EDTA-free protease inhibitors). After centrifugation, the supernatant was incubated with antibody at 4°C for 90 min, followed by incubation with protein A/G-agarose for another hour. Immunocomplexes were washed four times in lysis buffer and twice with kinase buffer (25 mM HEPES, pH 7.5, 100 mM potassium acetate, 2 mM MgCl_2_). The immunocomplexes were terminated by adding 10 μl of 4× SDS sample buffer, followed by Western analysis.

### Statistical analysis

All experiments were repeated at least three times using independent culture preparations. All measurements were performed blindly. The significance of difference between means was analyzed by the ANOVA and post hoc Bonferroni/Dunn tests (for multiple comparisons) and by the Student's *t* test (for single comparisons). The results are represented as means ± SEM. Statistical significance was determined by a value of *p* < 0.05 for all analyses.
